# Repetitive ultramicrotome trimming and SEM imaging for characterizing printed multilayer structures

**DOI:** 10.1038/s41598-024-79717-0

**Published:** 2024-11-20

**Authors:** Liyu Huang, Tim P. Mach, Joachim R. Binder, Richard Thelen, Ronald Curticean, Irene Wacker, Rasmus R. Schröder, Ulrich Gengenbach

**Affiliations:** 1 Institute for Automation and Applied Informatics, Karlsruhe Institute of Technology, Eggenstein-Leopoldshafen, 76344 Germany; 2 Institute for Applied Materials, Karlsruhe Institute of Technology, Eggenstein-Leopoldshafen, 76344 Germany; 3 Institute of Microstructure Technology, Karlsruhe Institute of Technology, Eggenstein-Leopoldshafen, 76344 Germany; 4 BioQuant, Universität Heidelberg, Im Neuenheimer Feld 267, Heidelberg, 69120 Germany

**Keywords:** Ultramicrotomy, Inkjet printing, Multilayer, SEM imaging, Characterization and analytical techniques, Electrical and electronic engineering, Nanoparticles, Nanoparticles, Scanning electron microscopy

## Abstract

Ultramicrotomy is a well-established technique that has been applied in biology and medical research to produce thin sections or a blockface of an embedded sample for microscopy. Recently, this technique has also been applied in materials science or micro- and nanotechnology as a sample preparation method for subsequent characterization. In this work, an application of ultramicrotomy for the cross-section preparation of an inkjet-printed multilayer structure is demonstrated. The investigated device is a capacitor consisting of three layers. The top and bottom electrodes are printed with silver nanoparticle ink and the dielectric layer with a ceramic nanoparticle/polymer ink. A 3D profilometer is initially used to study the surface morphology of the printed multilayer. The measurements show that both electrodes exhibit a coffee-ring effect, which results in an inhomogeneous layer structure of the device. To obtain precise 3D information on the multilayer, cross-sections must be prepared. Argon ion beam milling is the current gold standard to produce a single cross-section in good quality, however, the cross-section position within the multilayer volume is poorly defined. Moreover, the milling process requires a significant investment of time and resources. Herein, we develop an efficient method to realize repetitive cross-section preparation at well-defined positions in the multilayer volume. Repetitive cross-sections are exposed by trimming with an ultramicrotome (UM) and this blockface is subsequently transferred into a scanning electron microscope (SEM) for imaging. A combination of custom-modified UM and SEM specimen holders allows repeated transfer of the clamped multilayer sample between instruments without damage and with high positioning accuracy. This novel approach enhances the combination of an established ultramicrotome and a SEM for multilayer sample volume investigation. Thus, a comprehensive understanding of printed multilayer structures can be gained, to derive insights for optimization of device architecture and printing process.

## Introduction

Analysis of multilayers on a micrometer down to nanometer scale is a cross-cutting task in many scientific and technical fields. In materials science, the grain structure of large 3D volumes of steel samples has been investigated with high spatial resolution with an automated polishing and SEM imaging process^1^. In these applications, high-end instruments allow for the investigation of large sample volumes with dedicated preparation techniques and a substantial investment of resources. Cross-section analysis is also a frequent task in silicon integrated circuit (IC) analysis for studying the multilayer structure of the IC applied for quality control or as part of reverse engineering processes^2^. Cross-section preparation of silicon ICs is a multistage process. As silicon is a brittle material the chip to be analysed is either scribed and broken or cut with a diamond saw. The resulting coarse cross-section is ground and polished with an abrasive slurry (e.g. silicon carbide, diamond grains) in several steps or ion milled to obtain a good-quality cross-section surface for SEM imaging^3^. For inspection of small chip cross-section regions preparation by focused ion beam (FIB) milling and subsequent scanning electron microscope (SEM) imaging are applied. The same questions as in silicon ICs arise in the analysis of multilayer structures in printed electronics. However, in printed electronics, the substrate on which the devices are fabricated frequently is not a hard and brittle material, such as silicon, but rather a soft polymer foil such polyethylene terephthalate (PET) or polyimide (PI). For this material combination ultramicrotomy, a cross-section preparation process applied for soft samples from biology and medicine can be applied.

An Ultramicrotome (UM) is a precision instrument widely applied in biomedical research to produce (ultra)thin sections with a thickness as low as tens of nanometers^4–6^. A diamond knife is commonly used to achieve high-quality thin sections^7^. The ultramicrotome uses an orthogonal cutting mechanism and has demonstrated great versatility in preparing a large variety of samples^8–13^. This technique allows the investigation of fine structural details of cells or tissues, facilitating comprehension of their functional mechanism in relationship to structure^14–16^. The complete sample preparation process utilizing an UM is mainly empirical and subject to variations depending on sample properties, preparation techniques, tools employed, process parameters, and other factors^4,17–21^. The technique of ultramicrotomy has generated significant interest in the materials science field in the last years. Multiple reports have documented the successful utilization of this technique on a variety of materials for the purpose of structural characterization^22,23^. However, it should be noted that there still remain numerous challenges associated with applying this technique to material science samples^19,21^. An interesting application field is the structural investigation of printed multilayer passive (e.g. capacitors) and active (e.g. transistors, photovoltaics, organic light emitting diodes (OLED)) devices^24–26^. Here, the structural investigation of the multilayer volume is of utmost importance for the optimisation of the device architecture and the printing process.

This work demonstrates an application of UM and SEM imaging for structural investigation of a printed three-layer device. With the emergence of printed electronics, the fast fabrication of low-cost electrical elements on a variety of substrates has been made available through different techniques such as inkjet-printing, screen-printing and flexographic printing^27,28^. Inkjet-printing has been established as a non-contact method with low material waste and the ability of printing complex patterns at high resolution^28,29^. With this process, functional inks can be used to fabricate devices with tunable dielectric properties of the printed dielectric layer of a capacitor for example by deposition of non-linear ferroelectrics. These types of devices can be utilized as so-called varactors, which are voltage-tunable capacitors, characterized by their ability to change their relative permittivity under the application of an external electric field. They are applied in microwave devices such as phase shifters^30^, impedance tuners^31^, and for antenna beam steering^32^. In order to optimise the capacitor architecture and fabrication process detailed volume information on layer thickness and material homogeneity is required. Thus a capacitor device is used as a case study for the novel repetitive UM sectioning and imaging process.

**Fig. 1:**
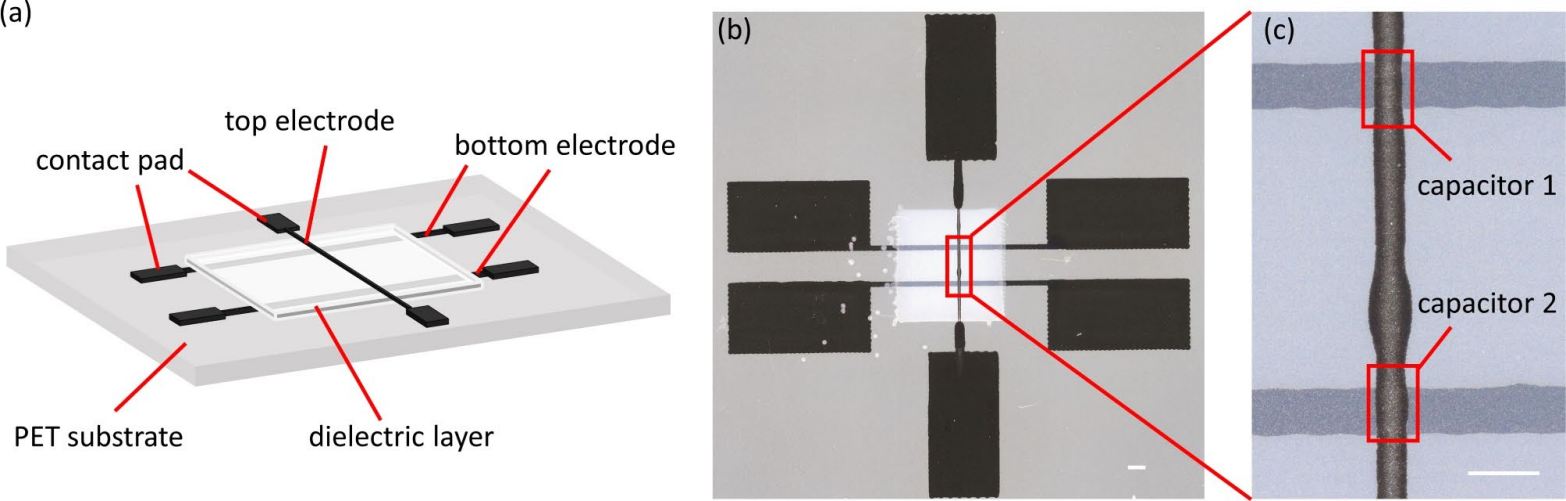
The printed capacitors: (**a**) a 3D schematic of the printed multilayer structure with six contact pads, three conductive lines and a 3 x 3 mm square dielectric layer; (**b**) top view captured with an optical microscope of the printed multilayer structure; (**c**) enlarged regions of the two capacitors at the intersection of the top and bottom electrodes. Scale bars: (**b**) $500\,\mu \hbox {m}$, (**c**) $200\,\mu \hbox {m}$.

As illustrated in Fig. 1a, the complete printed layout consists of six contact pads, three conductive lines and a square dielectric layer. The contact pads and electrodes are printed with silver nanoparticle ink whereas the interjacent dielectric layer is printed with a ceramic nanoparticle/polymer ink developed at KIT. The stack of three layers yields two capacitors as shown in Fig. 1b,c. The surface area of the printed device can be investigated directly by e.g. optical microscopy, whereas the interior structure of the multilayer is not easily accessible.

To facilitate such 3D examination of multilayers, volume electron microscopy, as applied in biological research, can be utilized. The biological sample is typically embedded in a medium, such as polymerized epoxy resin, to achieve mechanical stiffness for subsequent handling. The resulting sample block can then be investigated using microscopy. There are two principal methods for studying the sample structure: One is to expose the targeted region in the sample as a newly prepared block face, and the other is to cut and collect thin sections of the sample block, which contain a slice of the sample in each section^4^. Serial imaging of thin sample sections or block faces is an effective method for studying the 3D structure of diverse biological samples^15,33–35^. The sample is prepared using a variety of techniques, including ultramicrotomy or FIB milling, for subsequent SEM imaging. A FIB-SEM is a highly integrated instrument that typically uses a gallium ion beam to mill the sample and then image the resulting block face. However, the volume that can be analyzed in a single nanotomography run in a typical commercial instrument is limited to a few tens of microns in each direction. In addition, substantial damage in soft materials may be induced even using light elements, such as argon, neon or helium^36,37^. In contrast to FIB-SEM, the ultramicrotome can be used to trim the sample block face or to cut thin sample sections for microscopy. Obtaining serial thin sections is challenging, as there is a risk of damage to the sample structure during sectioning or section collection process. As an alternative, imaging the resulting sample block face can be a viable option. In particular, it has been demonstrated that an SEM integrated with a UM can automate the preparation and imaging of hundreds or even thousands of serial sample block faces^38^. However, studying the internal structure of printed multilayers frequently requires the preparation of only a few cross-sections at a few well-defined positions within the sample. Therefore, there is a need for an efficient and flexible method.

In this study, the surface profile of printed capacitors is initially examined with a 3D optical profilometer to quantitatively analyze the print quality by generating a top view of the dielectric layer. The surface roughness is calculated from these measurement data, indicating the dielectric layer’s print quality. Two cross-section preparation methods (Argon ion beam milling and UM trimming) are compared in detail with respect to positional targeting, process complexity, time consumption, and the quality of the resulting cross-sections. The thicknesses of the electrode layers and the dielectric layer are calculated from SEM images of cross-sections using ImageJ. This study demonstrates a novel application of ultramicrotomy in microtechnology, extending beyond traditional research areas such as (bio)medical technology.

## Results and discussion

This section presents the results and discussion of the experimental investigations, including the surface profile analysis conducted with a surface profilometer, the measurements and analysis of the prepared cross-sections, and a quality evaluation of cross-sections prepared by two different preparation techniques.

### Surface profilometry

**Fig. 2:**
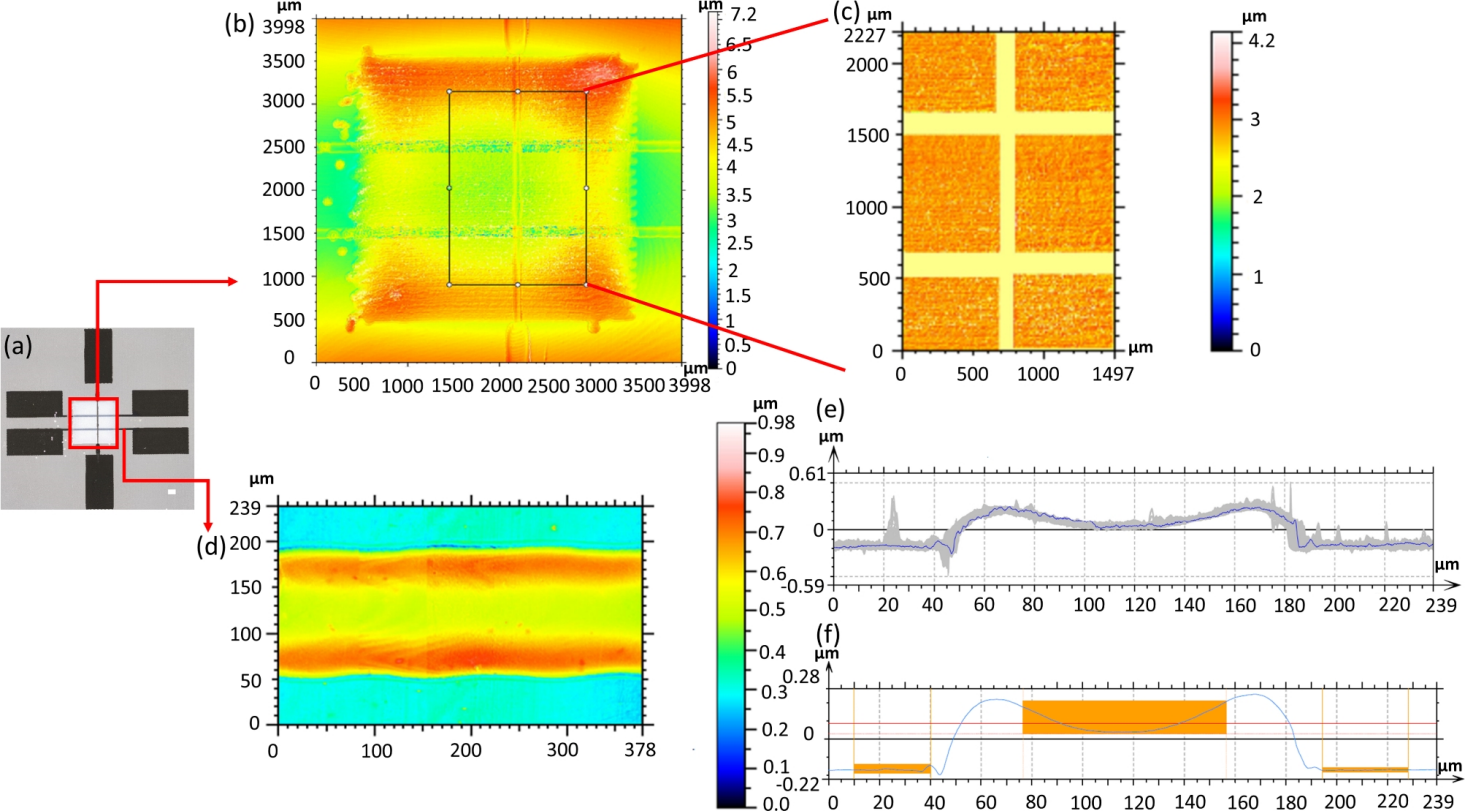
Surface profile analysis of the printed structure: (**a**) the regions marked in red are investigated for surface quality. Scale bar: $500\,\upmu \hbox {m}$; (**b**) overview of the scanned sample surface region as marked with a square in (**a**); (**c**) the surface roughness determined by white light interferometry of the marked region on the dielectric layer is $0.12\,\upmu \hbox {m}$; (**d**) surface topography of a section in the bottom electrode; (**e**) all available profiles across (as indicated by the grey bar) the bottom electrode are integrated in a diagram using the MountainsLab software; (**f**) the average profile across the bottom electrode is calculated by MountainsLab. The regions marked in orange are used to determine the electrode thickness. The red horizontal line across the upper orange region represents the average height of the marked region on the electrode surface. The distance between this red line and the substrate surface (the average height of the two lower orange regions) represents the average thickness of the bottom electrode. The thickness measured is $0.26\,\upmu \hbox {m}$. In (**e**, **f**) different scaling of the y axis.

Figure 2 illustrates the result of surface roughness measurements of a sample by white light interferometry. The entire square area was scanned to provide an overall image of the printed dielectric layer. The dielectric layer was printed line by line, the printing direction being horizontal in the image. At the vertical edges, the disconnected drops show that the edge quality is inferior to that of the edges parallel to the printing direction. This indicates that the print nozzle occasionally malfunctioned while toggling between the end of one line and the start of the next line. Additionally, areas near the dielectric layer edge are higher than the area inside, which suggests that the jetted ink exhibits a coffee-ring effect during the drying process (cf.^39^). A rectangular region with dimensions of $2500\,\upmu \hbox {m}\times 1250\,\upmu \hbox {m}$ enclosing the location of both capacitors is selected for surface roughness measurement. The electrode region is excluded from the data, to limit the roughness calculation to the printed dielectric layer. The arithmetic mean height of surface roughness ($S_a$) of this region of the dielectric layer is $0.12\,\mu \hbox {m}$. This measurement was conducted for nine samples from the same printing batch. As a result, $S_a$ varied from 0.11 to $0.16\,\upmu \hbox {m}$ (see also Table 1 in Supplementary Information). It indicates that the region close to the capacitor has a smaller surface roughness than the surrounding region in the complete square area. This evidence supports the conclusion that the printing strategy applied to ensure a good thickness homogeneity in the capacitor region is effective.

In addition to the measurement of the surface roughness of the printed dielectric, the profile of the bottom electrode on the substrate is also investigated using the same surface profilometer. As indicated in Fig. 2d, a section of the bottom electrode was scanned to investigate its profile. Using the software MountainsLab, in the scanned region, all profiles collected across the electrode are plotted in a diagram (Fig. 2e), and an average profile is calculated (Fig. 2f). The profile exhibits an obvious saddle-like outline, indicating that the printed electrode has elevated rims at both edges. This indicates a coffee ring effect along the length of the electrode. To calculate the thickness of the bottom electrode, the two peaks in the profile representing these elevated rims are excluded. Instead, the height differences between the substrate and the central region are measured to calculate the electrode thickness. The measured thickness is $0.26\,\upmu \hbox {m}$. A similar profile measurement was conducted along the bottom electrode to examine the thickness homogeneity. The maximum deviation in thickness along the bottom electrode is about $\pm 20\,\hbox {nm}$ or $\pm 7.7\%$ (see also Fig. S1 in Supplementary Information). These profile measurements demonstrate that the thickness along the bottom electrode is rather homogeneous, allowing the thickness measured outside of the capacitor region to serve as a thickness reference for the entire bottom electrode. Inspection of the measurement data of the top electrode indicate also a coffee-ring effect. Due to the roughness of the dielectric layer below, precise measurements and profile derivation are not feasible with this profile measurement method.

The results of the surface profile measurements show that the bottom electrode exhibits a significant coffee ring effect, which could affect the dielectric layer printed onto the bottom electrode. This results in an inhomogeneous thickness of the dielectric layer. The supposed crossing coffee-ring effect of the top and bottom electrodes leads to a complex layer geometry of the capacitor. These effects necessitate further optimization of the printing process to ensure that the parallel plate capacitor architecture is fabricated as designed. To gain more precise information on the inner structure of the printed multilayers, it is necessary to conduct a direct measurement of the capacitor cross-section.

### Preparation of cross-sections by argon ion beam milling

The blockface milled with the argon ion beam is free of contamination and the printed layers are clearly visible and distinguishable. The quality of the prepared cross-sections of the three samples is sufficient based on criteria (such as the absence of delamination, deformation, or contamination) indicating that the argon ion beam milling approach has good reproducibility (see also Fig. S3 in Supplementary Information). The bottom electrode, directly printed onto the substrate, exhibits a straight edge, while the top electrode printed onto the dielectric layer, displays a more undulated edge. This shows that the PET substrate has a significantly lower surface roughness than the printed dielectric layer. The thickness of the printed dielectric layer can be quantified using ImageJ by measuring the width between two detected edges (see also Fig. S2 in Supplementary Information). As the field of view is rather small under an SEM magnification of $5000 \times$, six images are taken to cover the entire length of the bottom electrode (see also Fig. S3 in Supplementary Information) to illustrate the thickness variation along the whole length. The SEM images and the thickness measurements both indicate that the printed dielectric layer exhibits an inhomogeneous thickness over its length.

Preparing one cross-section by argon ion beam milling takes over three hours. The manual handling of the sample and the alignment with the cover glass (cf. “Materials and methods”) also impede further milling processes to produce more cross-sections at well-defined positions over the width of a capacitor. Hence, this process is limited to single cross-section preparations at a certain depth in the capacitor.

### Cross-sections prepared by repetitive UM trimming

**Fig. 3:**
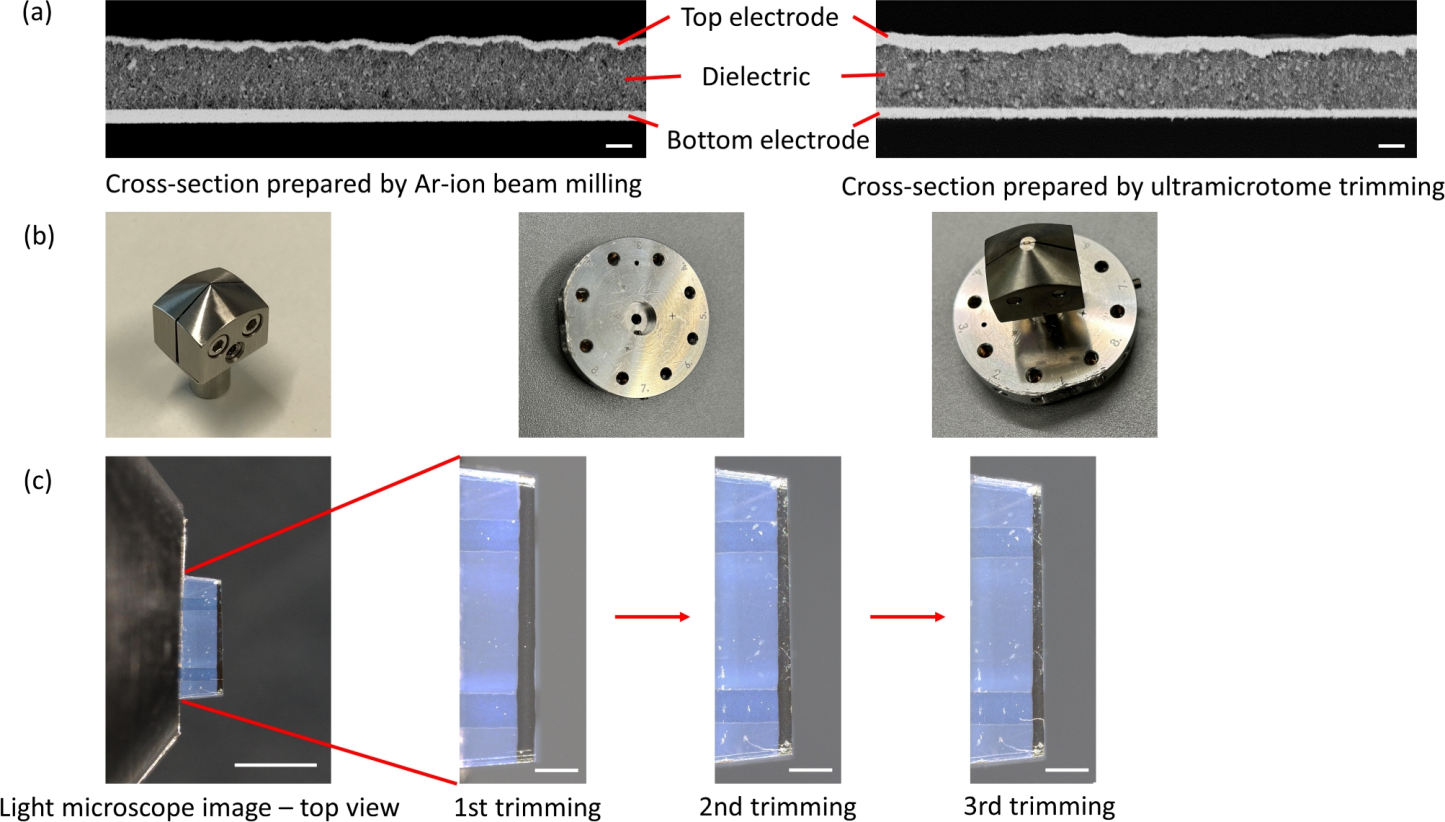
Utilizing an ultramicrotome to produce cross-sections of the printed multilayer structure for subsequent SEM imaging: (**a**) cross-sections prepared by Ar-ion beam milling and ultramicrotome trimming viewed in an SEM, scale bar: $1\,\upmu \hbox {m}$; (**b**) a non-magnetic flat sample holder for UM (left), the tip of the clamping jaws is trimmed (right) to create a flat opening, and the central borehole of the SEM specimen holder is enlarged to 9.85 mm (center) to mount the UM flat sample holder, diameter of SEM specimen holder, 46 mm; (**c**) the sample is fixed in the UM holder for three times repetitive trimming and SEM imaging, scale bar in top view: 1 mm, in images of sample trimming: $250\,\upmu \hbox {m}$.

Ultramicrotome trimming is an alternative process for cross-section preparation. Figure 3a shows cross-sections prepared by argon ion beam milling and UM trimming in comparison. Both cross-sections are free of contamination, delamination or deformation, indicating that neither process causes considerable damage to the printed multilayer structure. Thus, both methods are suitable for preparing cross-sections of samples of this kind. To realize a repetitive cross-section preparation the manual sample handling steps, in particular transferring the sample between different sample holders, have to be eliminated. Upon application of an UM for cross-section preparation, a universal holder for film specimens usable for both UM trimming and SEM imaging is desirable. However, there is no such product available on the market. Hence, we combine and optimize a currently available UM sample holder and an SEM specimen holder. This is illustrated in Fig. 3b. A non-magnetic UM flat sample holder (Electron Microscopy Sciences, USA) was selected to avoid interference with the electromagnetic fields inside an SEM. To facilitate the fixation of a flat sample, the original clamping jaws with a tip are cut off to create a flat opening for the sample. The film sample with the printed capacitor is fixed in this holder for trimming. To make the sample holder directly applicable for SEM imaging, the central borehole of an SEM specimen holder (Carl Zeiss Microscopy Deutschland GmbH, Germany) is enlarged to 9.85 mm, which is the diameter of the cylindrical shaft of the UM flat sample holder. In this way, the UM flat sample holder can be directly mounted onto the SEM specimen holder for subsequent SEM imaging. After imaging, the UM sample holder can be removed from the SEM specimen holder and mounted to the UM again for further trimming. Hence, the critical manual steps of repeatedly clamping and unclamping the delicate film sample are eliminated. This optimization allows the sample to be fixed only once in the UM holder, maintaining its position throughout the characterization process, which enables repetitive sectioning and SEM imaging with defined increments between the successive cross-sections.

**Fig. 4:**
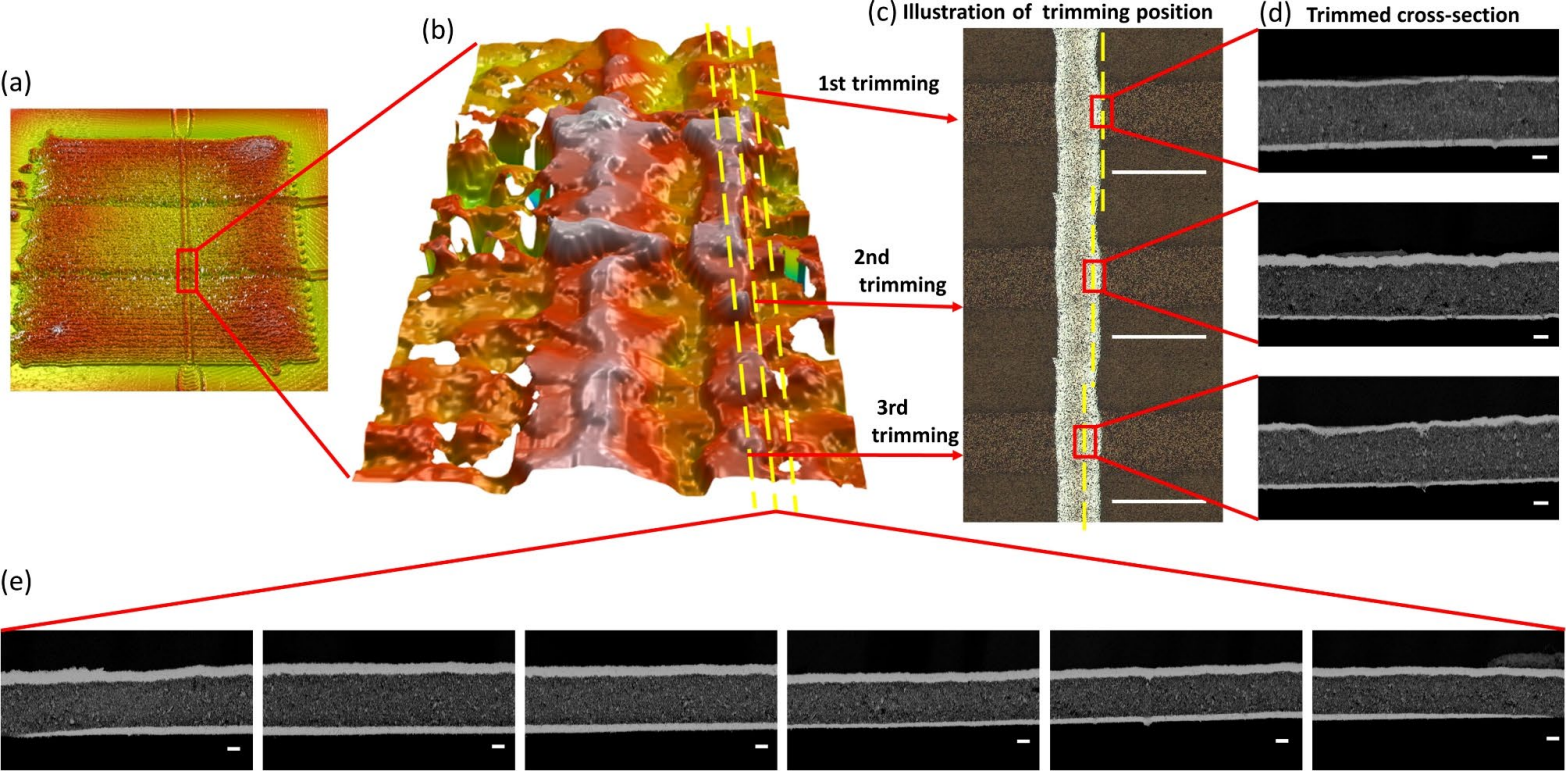
Illustration of the repetitive UM trimming and SEM imaging process with correlation to the 3D profilometer data: (**a**) an overview of the scanned surface of the printed dielectric layer and the top electrode; (**b**) a 3D illustration derived from profilometer measurement data. The yellow dashed lines represent the approximate trimming positions; (**c**) illustration of the approximate trimming positions in the top view; (**d**) SEM images are taken in the center of the obtained cross-sections after each trimming; (**e**) six images are taken along the length of a capacitor from the cross-section of the second trimming. Scale bar: (**c**) $200\,\upmu \hbox {m}$, (**d**, **e**) 1 $\upmu \hbox {m}$.

The left-most image of Fig. 3c shows a top view of a small piece of film with two printed capacitors clamped into the modified UM sample holder and trimmed close to the edge of the top electrode. The enlarged light microscope images illustrate the top view of the sample after each cross-section trimming. The sample was trimmed three times along the width of the top electrode as further shown in Fig. 4. The first trimming step was manually controlled to reach the edge of the electrode on the dielectric layer. Further trimming was performed with a defined trimming depth of ten microns each (Fig. 4b,c). The SEM images of the trimmed cross-sections showed clean surfaces after each trimming. No discernible damage was observed, and the printed multi-layer structure is clearly visible, indicating that this method yields comparable results to argon ion beam milling (Fig. 4d). While the bottom electrode shows only a small variation in thickness across the three sections, the dielectric and the top electrode show substantial thickness variations. Surface profilometer measurement data led to also suspect a coffee ring effect in the top electrode (Fig. 4a,b). It is, however, not clearly discernible due to the measurement noise. The SEM images of sections in Fig. 4d) cut along the rim of this second coffee ring effect (yellow lines in Fig. 4b) confirm this notion. As illustrated in Fig. 4d, the thickness of the top electrode exhibits a notable variation following multiple trimmings. Initially, the electrode is thin after the first trimming, subsequently becoming thicker after the second trimming, and then thinning once more after the third trimming. This observed trend correlates well with the corresponding profilometer measurement data presented in Fig. 4b. Figure 4e shows a sequence of six SEM images across the bottom electrode. It becomes evident that the electrode thickness is not constant but the peripheral regions are elevated, and the central region exhibits a lower thickness. This is in line with the profilometer measurements, indicating a coffee-ring effect of the bottom electrode. Using our repetitive UM trimming technique, three-dimensional structural information about the printed multilayer can be obtained. Our findings demonstrate that both electrodes display a coffee-ring effect and that the central dielectric layer is not homogeneous. Hence, these UM/SEM investigations clearly show that due to the printing parameters selected for the fabrication of this sample, the resulting multilayer stack geometry does not match the intended parallel plate capacitor architecture.

Besides its potential for investigating the 3D structure of a printed multilayer our UM/SEM method offers the advantage of reducing sample handling. The sample remains securely fixed in the holder following the initial clamping process for both trimming and SEM imaging, thereby facilitating precise positioning and control during the trimming process. Depending on the user’s experience, if the sample is properly aligned with the knife cutting edge in the UM, the discrepancy between the defined trimming depth and the actual trimming distance should be less than one UM arm feed (500 nm in this study). In comparison to argon ion beam milling, UM trimming is a significantly faster process, requiring approximately 20 minutes to prepare a single cross-section. Due to the repetitive nature of the trimming process, it allows for the preparation of cross-sections at well-defined varying lateral positions in the multilayer. This enabled the acquisition of comprehensive information on its volume after three trimmings.

### Evaluation of both cross-section preparation techniques regarding potential sample structure damage

To assess potential structural deformation such as compression or delamination resulting from the two cross-section preparation processes, the thickness of the bottom electrode is quantified using identical algorithms in ImageJ (see also Fig. S4 in Supplementary Information) and compared to the results of profilometer measurements. The profilometer measurements revealed that the bottom electrode exhibited a pronounced coffee ring effect, resulting in a saddle-like profile across the electrode as shown in Fig. 2e. In this comparison, we select the central part of the profile for the thickness measurements. The thickness of the bottom electrode is $0.265 \pm 0.053\,\upmu \hbox {m}$ after argon ion beam milling and $0.266 \pm 0.078\,\upmu \hbox {m}$ after UM trimming (see also Fig. S4 in Supplementary Information), which is comparable to the thickness of $0.26\,\upmu \hbox {m}$ obtained from the optical profile measurement (see Fig. 2f). These measurements and visual inspection of the cross-sections indicate that both cross-section preparation techniques do not cause structural deformation or even delamination of the samples.

## Conclusion

Our multilayer use case in this work, a printed capacitor, consists of layers with disparate material properties. In 3D this stack of layers is only held together by adhesion. To investigate the 3D structure of a multilayer of this kind, blockface imaging, a widely applied method in biomedical research, was adopted. It is imperative to prepare a sample cross-section at defined positions free from damage to the multilayer such as delamination, deformation or damage to its molecular structure. FIB-SEM imaging is not applicable due to its limited sample volume and the potential structural damage to the sample. Properly clamped, the multilayer exhibits sufficient mechanical stiffness to withstand UM trimming with a diamond knife without adverse effects to its blockface. Hence, sample embedding is not required.

This study introduced a novel method for cross-section preparation and SEM imaging and its application for the investigation of an ink-jet printed multi-layer structure. For the initial investigation of the printed capacitor, a 3D optical profilometer was applied to scan the top surface of the printed dielectric layer. This method is limited to the investigation of the top surface of the printed dielectric layer. High-resolution topography information within the multilayer volume can be acquired by direct measurement on cross-sections, where the interfaces between printed layers can be observed. This work compares two cross-section preparation techniques. Argon ion beam milling allows the preparation of single cross-sections of printed multilayers in high quality, although the required time (3 h) is considerable and the position of the cross-section within the investigated multilayer volume is poorly defined. Our novel repetitive UM trimming and SEM imaging method, based on a modified UM sample holder and SEM specimen holder assembly, offers a convenient approach for preparing multiple cross-sections at well-defined positions in the multilayer volume. This method overcomes the disadvantage of repeated sample change between different holders, as the sample is clamped only once with the new modified holders. This enables precise repositioning of the sample for both, UM trimming and SEM imaging, thus facilitating a repetitive trimming and imaging process cycle. Furthermore, the trimming process requires approximately 20 min, far less than the time required for argon ion beam milling. Unlike more sophisticated approaches relying on expensive instrumentation or even dedicated custom-built setups, it offers a new and easily accessible way for investigation of large volumes of e.g. printed multilayers with standard lab instrumentation such as an ultramicrotome and an SEM. Further studies may be conducted using a three-dimensional reconstruction based on an SEM image stack of repetitively trimmed and imaged cross-sections. The resolution in the trimming depth direction may be enhanced by reducing the step length between trimmings.

## Methods

This section presents the materials and methods applied in this study, including the optical investigation of the sample surface with a surface profilometer, preparation of sample cross-sections by means of argon ion beam milling and repetitive UM trimming, and the imaging process in an SEM. Furthermore the fabrication of the multilayer sample, the use case in this study is outlined.

### Multilayer sample fabrication

The commercial silver nanoparticle (AgNP) ink (Silverjet DGP-40LT-15C, Sigma Aldrich, Taufkirchen, Germany) is used to print the electrodes. The ceramic nanoparticle/polymer composite ink is developed in-house and consists of Ba_0.6_Sr_0.4_TiO_3_ with a particle size of d_99_ = 115 nm and poly(vinylidene fluoride-co-trifluoroethylene) (Solvene 200/P200, Sigma Aldrich, Taufkirchen, Germany) with a volume ratio of 1:1 and a total solid content of 4 vol.% in a solvent of dimethyl sulfoxide (DMSO) and methyl ethyl ketone (MEK) with a volume ratio of DMSO/MEK 1:1. A single nozzle piezoelectric drop-on-demand inkjet printer (Autodrop Professional; Microdrop, Norderstedt, Germany) with a printhead with $70\,\upmu \hbox {m}$ nozzle diameter and a printhead temperature of $\hbox {T} = 30^{\circ }\hbox {C}$ is applied to print the BST/P(VDF-TrFE) ink on polyethyleneterephthalate (PET) substrates (Melinex ST 506 films ($175\,\upmu \hbox {m}$), Dupont Teijin Films, Contern, Luxembourg). A driving voltage of U = 59 V and a pulse length of $67\,\upmu \hbox {s}$ are set at an ejection frequency of 500 Hz to obtain stable printing conditions. A vacuum of 10 mbar is applied to the ink vessel. Monitoring of drop formation took place by using a camera and a strobe diode with a delay time of 500 s. The ink is dried on the substrate table of the printing system, at $45^{\circ }\hbox {C}$. The silver electrodes are printed with AgNP ink using a printhead with a $50\,\upmu \hbox {m}$ nozzle. The bottom Ag-electrodes are printed on the heated substrate table at a temperature of $80^{\circ }\hbox {C}$. Afterwards, to yield a continuous conductive layer, the electrodes are sintered at $120^{\circ }\hbox {C}$ for 1 h. The dielectric layer is then printed on top and in a final step, the top Ag-electrode is printed with a substrate temperature of $80^{\circ }\hbox {C}$. The capacitor is dried in a vacuum drying oven at $90^{\circ }\hbox {C}$ for 20 h. Two electrode lines are printed as bottom electrodes to guarantee a high amount of working capacitors in case the printing process would leave electrode lines disconnected.

### Surface profile measurements of the printed multilayer

The printed dielectric layer surface, shown in Fig. 1b, lacks reflection due to its translucence, which adversely affects white-light interferometer measurements. To obtain a reflective surface, the sample is coated with a silver layer with a sputter coater (Cressington Sputter Coater 108auto, TESCAN GmbH, Germany) at 30 mA and 0.1 mbar for 60 s. The silver layer’s coated thickness is approximately 10 nm, which is negligible compared to the thickness of the dielectric layer. The complete surface of the printed dielectric layer (as indicated in red in Fig. 2a) is scanned with a 3D optical profilometer (ContourX-500, Bruker optics, Germany) to investigate the overall surface quality of the printed dielectric. Since the effective region of the capacitors is located between the printed electrodes, it is not directly accessible for roughness measurement. A larger area close to the capacitor, with dimensions of 1250 x 2500 microns (as indicated in red in Fig. 2b), is scanned in greater detail to measure surface roughness.

### Profilometer data analysis with MountainsLab

The analysis is conducted following the ISO 25178 standard for 3D surface texture in the software MountainsLab (Digital Surf, France). The regions of the printed bottom and top electrodes are identified and removed from the surface roughness measurement, as they are printed with a different ink (silver nanoparticle dispersion) and thus exhibit a different surface quality compared to the dielectric layer. The resulting area of the printed dielectric is utilized for the roughness analysis. The function ”all available profiles” of the MountainsLab software was applied to evaluate the thickness variation along the length of the bottom electrode. The 3D model as shown in the Fig. 4b at the region of capacitor 2 is obtained after levelling, form subtraction, and filtering with 5 x 5 pixels.

### Cross-section preparation with Argon Ion Beam Milling

The cross-section of printed multi-layers is prepared with a target preparation device (Leica EM TXP, Leica Microsystems, Germany), using a diamond saw at a speed of 15,000 rpm with a subsequent polishing process with a $5\,\upmu \hbox {m}$ SiC lapping foil at a speed of 2500 rpm. Afterwards, the sample is transferred and glued onto a Leica EM TIC 3X Al sample holder. A cover glass (100 µm thickness) is fixed on top of the sample surface to protect the structure from edge rounding by the ion beam. Then, the sample is further processed by an argon ion beam milling system (Leica EM TIC 3X Ion Beam Milling System, Leica Microsystems, Germany) with a voltage of 6 kV and a current of 2.2 mA for 3 h.

### Cross-section preparation with Ultramicrotomy

An UM (Leica EM UC7, Leica Microsystem, Germany) with a diamond knife (Trim 90, DiATOME, Switzerland) is utilized for precise cross-section trimming. The cutting speed is set to 6 mm/s, and the feed is adjusted to 500 nm based on previous tests to ensure a good cross-section quality for imaging.

### Investigation of the multilayer cross-sections in a SEM

A scanning electron microscope (SUPRA 55, Carl Zeiss Microscopy GmbH, Germany) is used to image the prepared sample cross-sections. The angle selective backscattered (AsB) detector is utilized to enhance the contrast of the printed multi-layers. An accelerating voltage of 10 kV, a beam current of $80\,\upmu \hbox {A}$ and a working distance of 5 mm were applied.

### SEM image analyis

The acquired images are processed in ImageJ with the macro “Analyze Stripes”, to detect the edges of the electrodes and the dielectric layer. Moreover, an average width between the two detected edges of the dielectric layer is calculated, representing the thickness. Hence, six measurements are taken respectively on each capacitor to obtain significant precision. The final thickness of the dielectric layer is the average of the six measurements.

## Supplementary Information

Supplementary Information.

## Data Availability

Additional information on the surface profile analysis and the thickness determination on the prepared cross-sections of the printed multilayer structures is summarized in the Supplementary Information (also referenced in the main text). The complete generated surface profile data and the original SEM images are available from the corresponding author on reasonable request.
