# Supplementary material for: Repetitive ultramicrotome trimming and SEM imaging for characterizing printed multilayer structures
Source: Sci Rep. 2024 Nov 20;14:28716. doi: 10.1038/s41598-024-79717-0 (PMC11579308; doi:10.1038/s41598-024-79717-0)
Supplement: Supplementary file 1 — Supplementary Information. [file 41598_2024_79717_MOESM1_ESM.pdf]

Supplementary information

Repetitive ultramicrotome trimming and SEM imaging for characterizing printed multilayer structures

Liyu Huang*, Tim P. Mach, Joachim R. Binder, Richard Thelen, Ronald Curticean, Irene Wacker, Rasmus R. Schröder, Ulrich Gengenbach

*liyu.huang@kit.edu

The supplementary information consists of one table and four images, referenced in the main text to provide additional relevant information about the experiments.

Sample number	S_a (μm)
1	0.15
2	0.12
3	0.12
4	0.12
5	0.12
6	0.16
7	0.12
8	0.11
9	0.13

Table 1. Dielectric surface roughness measurements of nine samples of printed multilayer capacitor devices

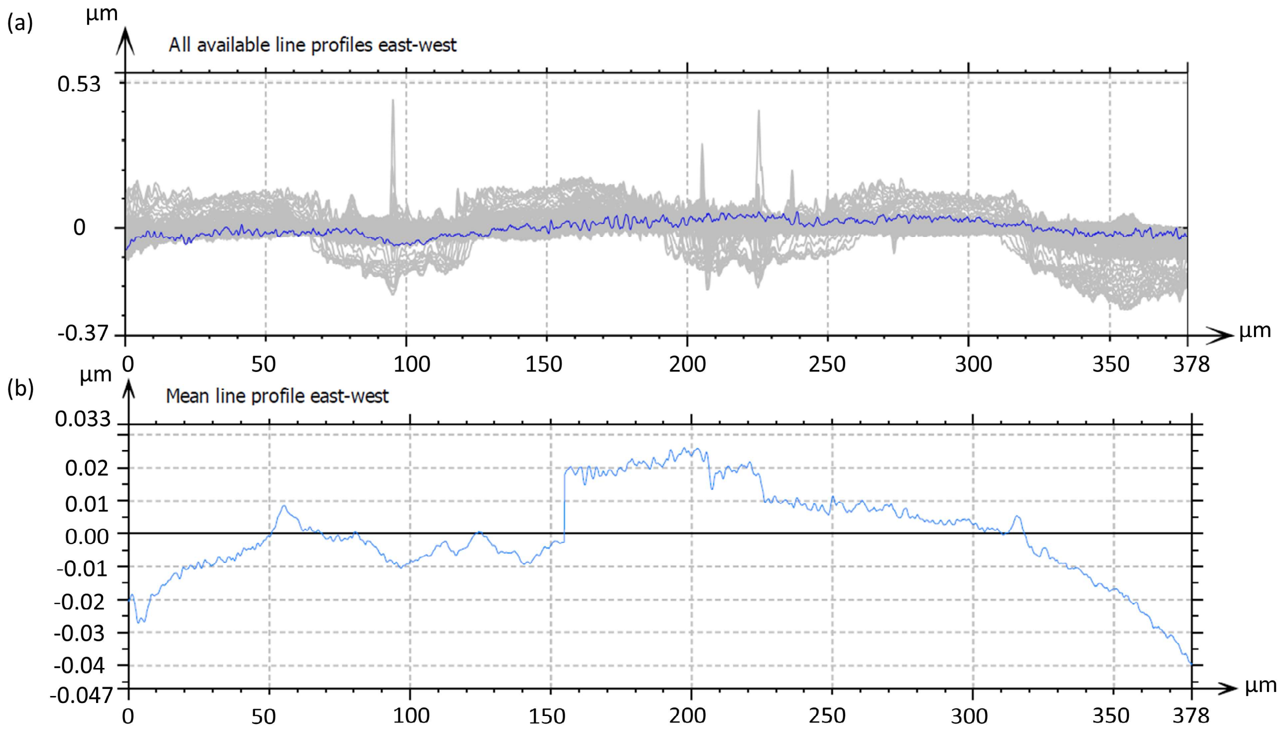

Figure S1. Surface profile along the length of the bottom electrode: (a) all available profiles are integrated into one diagram; (b) an average of all profiles is calculated (all measurement data evaluations with MountainsLab software).

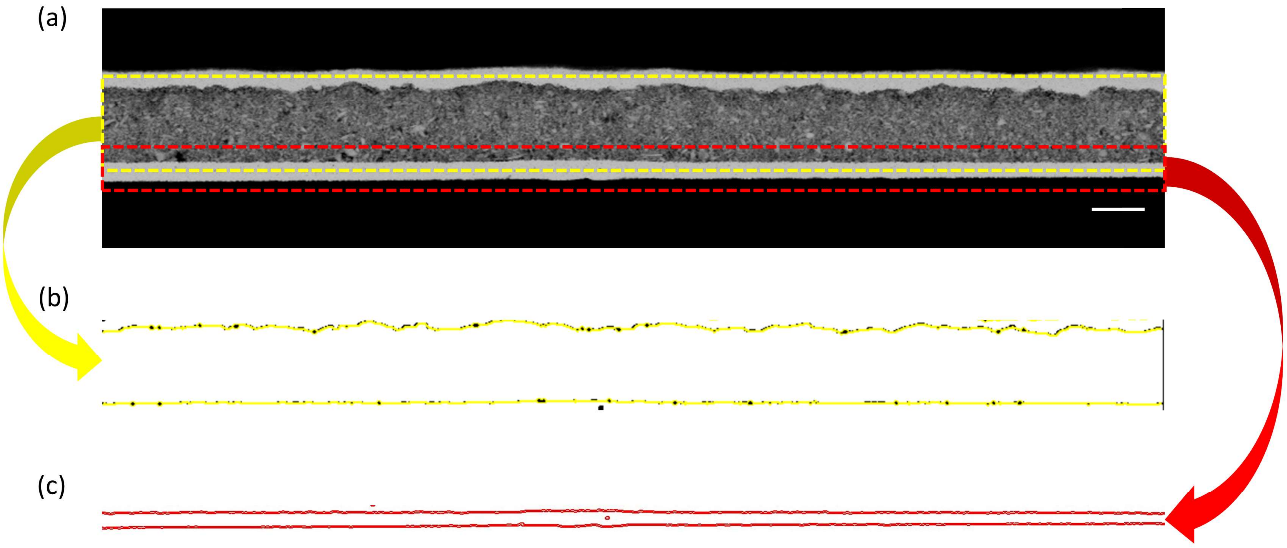

Figure S2. The average thicknesses between the detected two edges of the printed layers are calculated with ImageJ: (a) the region marked in yellow is used for the edge detection of the dielectric layer and the region marked in red for the edge detection of the bottom electrode; (b) edges detected by ImageJ of the dielectric layer; (c) of the bottom electrode. Scale bar: 1 μm.

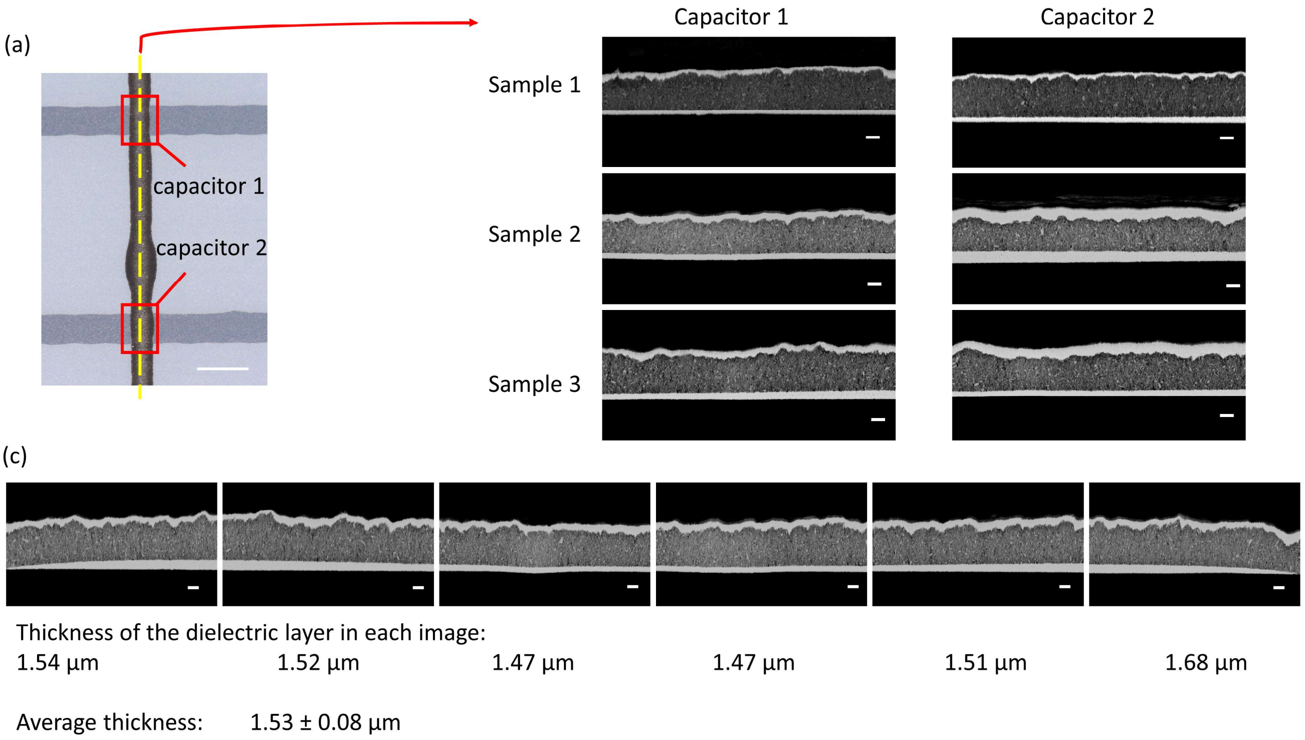

Figure S3. Cross-sections prepared by argon ion beam milling: (a) the position of the cross-section is manually adjusted to be approximately in the center of the top electrode (yellow line); (b) cross-sections of three samples imaged by SEM with an AsB detector; (c) cross-section of a bottom electrode assembled with six SEM images along the bottom electrode length, showing thickness variations due to the coffee-ring effect. Scale bar: (a): 200 μm, (b): 1 μm, (c): 1 μm.

(a)

Cross sections prepared by argon ion beam milling:

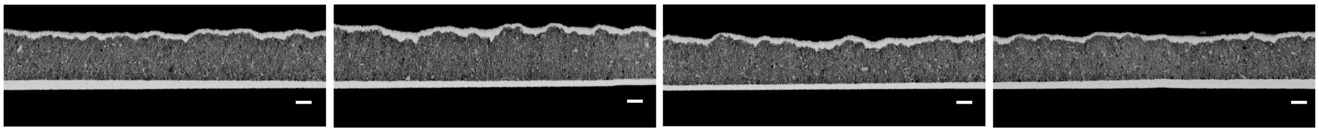

Thickness of the bottom electrode in each image:

0.314 µm   0.215 µm   0.208 µm   0.322 µm

Average thickness: 0.265 ± 0.053 µm

(b)

Cross sections prepared by UM trimming:

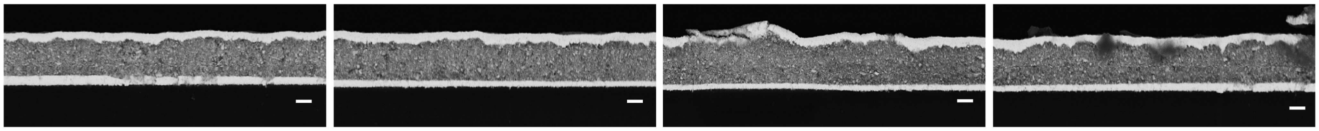

Thickness of the bottom electrode in each image:

0.379 µm   0.194 µm   0.193 µm   0.199 µm

Average thickness: 0.266 ± 0.078 µm

Figure S4. Comparison of the bottom electrode thickness after cross-section preparation with argon ion beam milling (a) and UM trimming (b). Both preparation processes were conducted on the same sample. To avoid the strong thickness variation caused by the coffee-ring effect in the bottom electrode, two sections at the edges are excluded from the thickness measurement, as in the profilometer measurement. The thickness is determined using ImageJ as shown in figure S2. Scale bar: 1 μm.
